# Activation of Interferon Signaling in Chronic Lymphocytic Leukemia Cells Contributes to Apoptosis Resistance via a JAK-Src/STAT3/Mcl-1 Signaling Pathway

**DOI:** 10.3390/biomedicines9020188

**Published:** 2021-02-13

**Authors:** Brigitte Bauvois, Elodie Pramil, Ludovic Jondreville, Claire Quiney, Florence Nguyen-Khac, Santos A. Susin

**Affiliations:** 1 Centre de Recherche des Cordeliers, INSERM, Cell Death and Drug Resistance in Lymphoproliferative Disorders Team, Sorbonne Université, Université Sorbonne Paris Cité, Université Paris Descartes, Université Paris Diderot, F-75006 Paris, France; elodie.pramil@hotmail.fr (E.P.); ludovic.jondreville@gmail.com (L.J.); claire.quiney@upmc.fr (C.Q.); florence.nguyen-khac@aphp.fr (F.N.-K.); santos.susin@crc.jussieu.fr (S.A.S.); 2 Assistance Publique-Hôpitaux de Paris, Groupe Hospitalier Pitié-Salpêtrière, Service d’Hématologie Biologique, F-75013 Paris, France

**Keywords:** apoptosis, CLL, IFN signaling, Mcl-1, STAT3, survival

## Abstract

Besides their antiviral and immunomodulatory functions, type I (α/β) and II (γ) interferons (IFNs) exhibit either beneficial or detrimental effects on tumor progression. Chronic lymphocytic leukemia (CLL) is characterized by the accumulation of abnormal CD5^+^ B lymphocytes that escape death. Drug resistance and disease relapse still occur in CLL. The triggering of IFN receptors is believed to be involved in the survival of CLL cells, but the underlying molecular mechanisms are not yet characterized. We show here that both type I and II IFNs promote the survival of primary CLL cells by counteracting the mitochondrial (intrinsic) apoptosis pathway. The survival process was associated with the upregulation of signal transducer and activator of transcription-3 (STAT3) and its target anti-apoptotic Mcl-1. Furthermore, the blockade of the STAT3/Mcl-1 pathway by pharmacological inhibitors against STAT3, TYK2 (for type I IFN) or JAK2 (for type II IFN) markedly reduced IFN-mediated CLL cell survival. Similarly, the selective Src family kinase inhibitor PP2 notably blocked IFN-mediated CLL cell survival by downregulating the protein levels of STAT3 and Mcl-1. Our work reveals a novel mechanism of resistance to apoptosis promoted by IFNs in CLL cells, whereby JAKs (TYK2, JAK2) and Src kinases activate in concert a STAT3/Mcl-1 signaling pathway. In view of current clinical developments of potent STAT3 and Mcl-1 inhibitors, a combination of conventional treatments with these inhibitors might thus constitute a new therapeutic strategy in CLL.

## 1. Introduction

From a biological point of view, interferons (IFNs) constitute a family of pleiotropic cytokines with antiviral and immunomodulatory functions [1,2,3]. IFNs are classified into three main types, including type I (mainly IFN-α and -β), type II (IFN-γ) and type III (IFN-λ, 1−4) [4,5,6,7,8]. Whereas type I and II IFNs are predominantly expressed by innate immune cells [1,2], type III IFNs are mainly produced by antigen-presenting cells and epithelial cells [8]. Type III IFN receptors are more restricted than type I and II receptors, and are mainly expressed by epithelial cells, macrophages and dendritic cells [3,8,9]. Type III IFNs are thought to maintain healthy mucosal surfaces through immune protection, with a decrease in immune-related pathogenic risk associated with type I IFN responses [8]. Current evidence highlights the paradoxical (i.e., both beneficial and detrimental) effects of type I and II IFNs on cellular processes associated with tumor development (proliferation, survival, invasion) [7]. Both type I and II IFNs signal through activation of receptor-associated Janus kinases (JAKs)/signal transducers and activators of transcription (STAT) pathways [4,5,6,7]. Aberrant STAT3 expression and activation (through phosphorylation) is observed in various solid and hematological tumors, and is associated with tumor growth and progression [10,11,12,13]. Type I and II IFNs promote specific phosphorylation of STAT3 at tyrosine^705^ (p^Y705^), allowing its dimerization, nuclear translocation, and transcriptional activation of target genes involved in cell proliferation, cell survival and metastasis [6,11,14]. Conversely, downregulation of STAT3 signaling inhibits tumor growth and induction of cell death in preclinical models [11,12]. Accumulating evidence suggests that STAT3 regulates resistance to chemotherapy and thereby impairs therapeutic efficacy [12].

Chronic lymphocytic leukemia (CLL) is a very heterogeneous disease characterized by a peripheral accumulation of abnormal CD5^+^ B lymphocytes in the blood, bone marrow and lymphoid tissues [15]. The leukemic cells (which are mostly quiescent) mainly accumulate because they are unable to develop a cell death program—even though proliferating pools are found in the bone marrow and lymph nodes [15]. Treatment of CLL remains a challenge in the clinic because ~15–25% of patients either are refractory to today’s front-line therapies or relapse after treatment [16]. The drugs currently prescribed are a combination of fludarabine-cyclophosphamide-rituximab (FCR), signaling inhibitors targeting B cell receptor (BCR)-associated kinases (i.e., Bruton’s tyrosine kinase (BTK) inhibitors such as ibrutinib) or the antagonist of the B-cell lymphoma-2 (Bcl-2) anti-apoptotic protein (venetoclax) [16]. Venetoclax has been initially approved for relapsed or refractory CLL patients [17], and today may be prescribed in first-line treatment of untreated CLL patients [18,19]. Unfortunately, these therapies are often accompanied by adverse effects or favored mutations associated to drug resistance [20,21,22]. Therefore, novel therapies are needed to overcome resistance to these drugs, and the identification of new therapeutic possibilities in CLL therapy is of general interest.

Higher amounts of STAT3 (mRNA, protein, phosphorylation status) are found in CLL cells as compared to normal B lymphocytes [23,24]. The CLL cells display specific receptors with high affinity for type I and II IFNs [25,26]. Although transcripts for type III IFN receptors are detectable in human B cell lineage cells [9], the protein expression of these receptors at the surface of normal and leukemic B cells remains to be demonstrated. Some data have shown that IFN-α and IFN-γ could protect CLL cells from spontaneous death [27,28,29,30], but the molecular mechanisms involved are not elucidated. In the present work, we report that type I and II IFNs trigger the survival of CLL cells by blocking the intrinsic apoptotic pathway. The underlying mechanisms and intracellular signaling pathways activated by IFNs were investigated, with a focus on STAT3 with regard to its key role in IFN signaling. Our results show the essential role of STAT3 in the type I/II IFN-mediated upregulation of the anti-apoptotic protein Mcl-1, and this might have implications in the context of the therapeutic potential of STAT3 and Mcl-1 inhibitors in CLL.

## 2. Materials and Methods

### 2.1. Ethics Statement

The study protocol was approved by the Ethicsl Committee on human experimentation at Pitié-Salpêtrière Hospital (21 May 2014, CPPIDF6, Paris, France). 

### 2.2. Patients and CLL Cell Separation

Peripheral blood was collected from 19 patients with CLL according to standard clinical criteria and the International Workshop on CLL (IWCLL) criteria [16] including lymphocyte morphology, Binet stage and *IGHV* mutational status. Deletions of 17p13, 11q22, 13q14 and trisomy 12 were detected using fluorescence in situ hybridization (FISH) with the Metasystems XL DLEU/LAMP/12cen and XL ATM/TP53 Multi-Color Probe Kits (MetaSystems, Compiègne, France). The biological and clinical characteristics of CLL patients are listed in Table 1. Peripheral blood mononuclear cells (PBMCs) were isolated from blood using Ficoll-Hypaque density gradient (1.077 g/mL) centrifugation. More than 90% of CLL PBMCs were CD19^+^CD5^+^. Freshly isolated cells were used immediately in culture assays. Cell pellets were frozen at −80 °C until RNA or protein extraction, and analysis.

### 2.3. Cell Culture Conditions

Freshly isolated PBMCs (10^6^/mL) were cultured in RPMI 1640 medium (Life Technologies, Paisley, UK) supplemented with 2 mM L-glutamine, 1 mM sodium pyruvate, and 40 μg/mL gentamicin, in a 5% CO_2_ humidified atmosphere at 37 °C. The cells were then treated with recombinant human IFN-αa (Hoffman-La Roche, Basel, Switzerland), -β (Ares-Serono, Geneva, Switzerland) or -γ (R&D Systems, Abingdon, UK) (1000 U/mL) for 18–30 h. In some experiments, cells were pretreated with a pharmacological inhibitor AG490 (10 μM; JAK2 inhibitor; Calbiochem, Darmsdat, Germany), AG9 (10 µM; TYK2 inhibitor; Calbiochem, Darmsdat, Germany), PP2 (10 µM; Src family inhibitor; Calbiochem, Darmsdat, Germany) or Stattic (5 µM; STAT3 inhibitor V; Calbiochem, Darmsdat, Germany) for 15 min prior to the addition of IFNs. For each pharmacological inhibitor, we applied the highest concentration that did not markedly affect the viability of CLL cells (i.e., a cell death rate of no more than 20%, relative to basal levels). In negative control experiments with inhibitors, cells were treated with the same volume of dimethyl sulfoxide alone. After incubation, the cells were collected, washed once, and then used for flow cytometry, DNA fragmentation, mitochondrial membrane permeability, active caspase-3 and ROS assays, RT-PCR and Western blot analyses.

### 2.4. Flow Cytometry

Intact CLL PBMCs were directly immunostained as previously described [31]. The balance between cell death and survival was assessed using the annexin V-FITC/propidium iodide (PI) cell death detection kit (Beckman-Coulter, Les Ullis, France). Intracellular active caspase-3 was detected using a specific FITC-conjugated rabbit IgG (C92-605, rabbit Ig; BD Biosciences, Le Pont de Claix, France) in cells permeabilized with the BD Cytofix/Cytoperm kit (BD Biosciences, Le Pont de Claix, France); the negative control was FITC-rabbit IgG (Santa-Cruz, Heidelberg, Germany). Mitochondrial transmembrane potential (ΔΨ_m_) was analyzed using the fluorescent dye cell permeant tetramethyl rhodamine ethyl ester (TMRE, 125 nM; Life Technologies Thermo Fisher, Illkirch, France). Mitochondrial ROS levels were detected in living cells using the MitoSOX dye (2 µM; Invitrogen, Paris, France) which reacts directly with ROS species, yielding a red fluorescence. Stained cells were analyzed with a Coulter Epics XL flow (Beckman-Coulter, Les Ullis, France) or a FACSCanto II flow (BD Biosciences, Le Pont de Claix, France) cytometer. Data were analyzed using LYSYS (Beckman-Coulter) or FloJo (BD Biosciences, Le Pont de Claix, France) software.

### 2.5. DNA Fragmentation Assay

DNA fragmentation (evaluated as by detecting an oligonucleosome ladder in agarose gel electrophoresis experiments) was assessed as described previously [32]. The DNA fragments were electrophoretically separated in 1.8% agarose gels containing 0.2 µg/mL ethidium bromide, and the gel bands were analyzed using a Quantum ST4 system (Vilber Lourmat, Marne La Vallée, France).

### 2.6. Real Time PCR Assays

RNA extraction from treated cells and cDNA synthesis were performed as described previously [31]. The cDNAs coding for human Mcl-1, STAT3, and β2-microglobulin were amplified in PCRs, using primers synthesized by Sigma-Proligo and Eurofins Genomics, according to the published sequences [31,33,34]. The PCR products were visualized by electrophoresis in a 1.8% agarose gel containing 0.2 µg/mL ethidium bromide. The bands were imaged in a Quantum ST4 system (Vilber Lourmat, Marne La Vallée, France). 

### 2.7. Immunoblotting

Cells were lysed in M-PER buffer (Pierce Biotechnology, Rockford, IL, USA) supplemented with protease and phosphatase inhibitor cocktails (Sigma). Total cell extracts were separated using sodium dodecyl sulfate-polyacrylamide gel electrophoresis (SDS-PAGE) in 10% gels, transferred to nitrocellulose membranes, and blotted as described previously [35]. The primary antibodies included anti-Mcl-1 (clone S-19, rabbit IgG; Santa-Cruz, Heidelberg, Germany), anti-Bcl-2 (clone 100, mouse IgG1; Santa-Cruz, Heidelberg, Germany), anti-STAT3 (clone C20, rabbit IgG; Santa-Cruz, Heidelberg, Germany), anti-p^Y705^-STAT3 (clone 710093, rabbit Ig; Thermo Fisher Scientific, Waltham, MA, USA), anti-Bax (6A7, mouse IgG; Santa-Cruz, Heidelberg, Germany), anti-Bak (Ab1, mIgG; Calbiochem, Darmsdat, Germany) and anti-actin (clone C4, mouse IgG1; ICN Biomedicals, Solon, OH, USA). Immunoreactive proteins were detected using horseradish peroxidase-conjugated secondary antibodies and visualized with the Pierce ECL Western blotting substrate system or the SuperSignal West Femto Maximum Sensitivity Substrate system (both from Thermo Fisher Scientific). In some experiments, removal of antibodies from Western blots was performed using the Millipore stripping solution (Molsheim, France). Immunoblot images were acquired in an MF-ChemiBIS v4.2 imager (DNR Bio-Imaging Systems Ltd., Neve Yamin, Israel) and quantified using ImageJ64 software (NIH, Bethesda, MD, USA).

### 2.8. Statistics

Statistical analyses were performed using GraphPad Prism software (version 7.0, GraphPad Software, La Jolla, CA, USA). Data were expressed as the mean ± standard error of the mean (SEM). Groups were compared using Mann–Whitney tests or unpaired or paired Student’s *t*-tests. For greater stringency, all tests were two-tailed. Significance levels were defined as * *p* < 0.05; ** *p* < 0.01; and *** *p* < 0.001.

## 3. Results

### 3.1. Type I and II IFNs Promote CLL Cell Survival by Counteracting the Intrinsic Apoptosis Pathway

**Figure 1:**
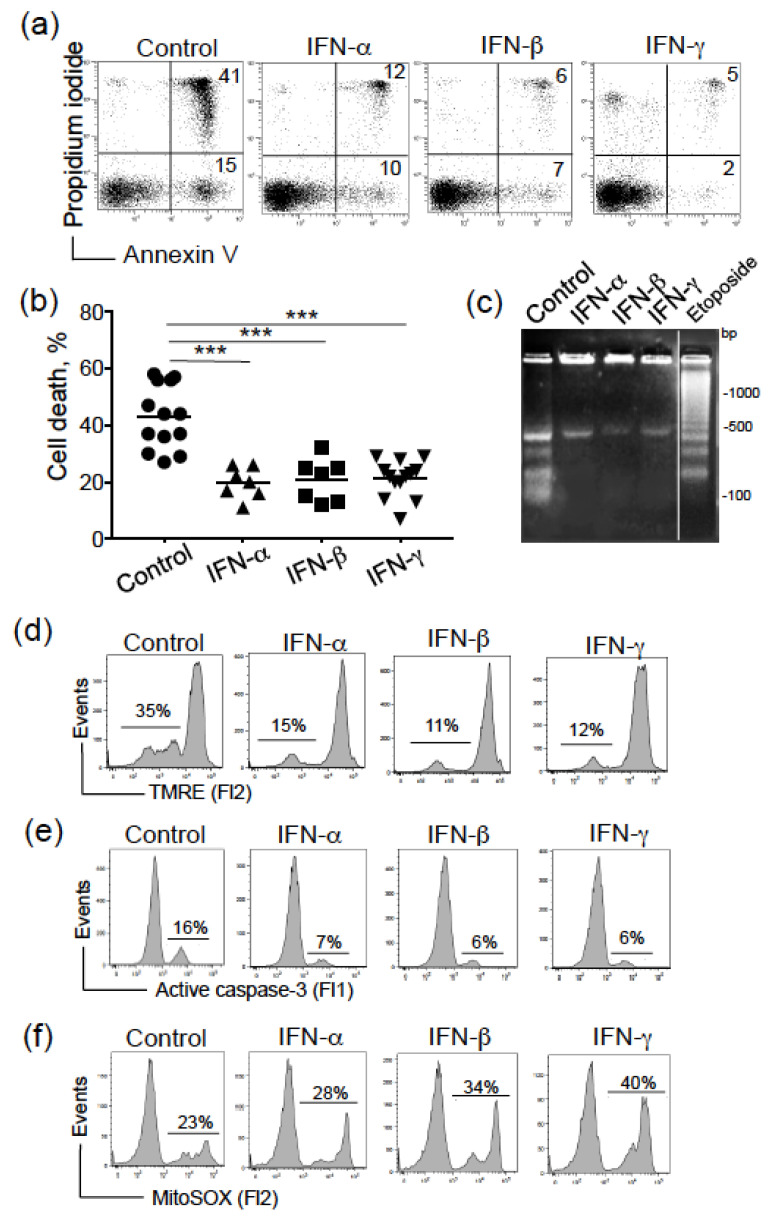
Type I and II IFNs induce resistance to apoptosis in CLL cells. (**a**) Representative cytograms of CLL cells cultured for 24 h in the presence or absence of IFN-α, -β, -γ (1000 U/mL); detection of apoptotic cells after annexin-V-FITC/PI staining and flow cytometry. The percentage of annexin-V-positive cells is shown. (**b**) The data of cell death are presented as mean ± SEM (13 controls, 7 treated IFN-α and -β, 13 treated IFN-γ). *P* values were calculated using the unpaired *t*-test. *** *p* < 0.001. (**c**) CLL cells were cultured for 24 h in the presence or absence of IFNs, then DNA fragmentation was evaluated by the detection of an oligonucleosome ladder by agarose gel electrophoresis; etoposide treatment (10 µM) was used as positive control of DNA fragmentation (**d**–**f**) CLL cells were cultured for 24 h in the presence or absence of IFNs (1000 U/mL, 24 h): (**d**) The ΔΨ_m_ was measured using the fluorescent probe TMRE, and analyzed by flow cytometry; the percentages refer to ΔΨ_m_ disruption. (**e**) Active caspase-3 expression was measured by flow cytometry; the percentages refer to the percentage of active caspase-3. (**f**) Mitochondrial ROS levels were recorded by flow cytometry with the use of MitoSOX. Percentages refer to cells with ROS levels.

We first examined the effects of type I (α, β) and II (γ) IFNs (1000 U/mL, for 24 h) on the viability of cultured CLL cells. Cell death was assessed by determining phosphatidylserine exposure at the cell surface (using annexin-V-FITC binding) and cell membrane disruption (using propidium iodide labeling). As exemplified in Figure 1a, the proportion of total annexin V^+^ cells (dead cells) was lower after treatment with type I or II IFNs than in control (untreated) experiments. The paired-t test confirmed the significant enhanced survival in IFN-treated CLL cells (Figure 1b). The protective effect of IFNs was independent of the Binet stage (stage A vs. stage B/C, *p =* 0.342). We further sought to determine whether or not IFNs could counteract the mitochondrial (intrinsic) pathway that controls the balance between cell death and survival in CLL [36]. Activation of the intrinsic apoptotic pathway provokes disruption of the mitochondrial transmembrane potential (ΔΨ_m_), caspase activation and DNA oligonucleosomal fragmentation [37,38]. Here, DNA fragmentation (<500 bp) at 24 h was already lower in IFN-treated CLL cells than in untreated cells (Figure 1c). The exposure of cells to IFNs for 24 h prevented ΔΨ_m_ disruption (evaluated as an increase in fluorescence intensity, relative to untreated cells; Figure 1d). In the process of apoptosis, caspase-3 is the ‘‘executioner enzyme’’ [39]. As expected, CLL cells treated with IFNs displayed lower levels of active caspase-3 than untreated cells (Figure 1e). The elevated levels of mitochondria-derived reactive oxygen species (ROS) correlate with CLL cell survival [40]. In a cell model of breast cancer, IFN-γ stimulates ROS-producing enzymes leading to mitochondrial ROS production [41]. In view of these data, we assessed the levels of ROS in IFN-treated CLL cells. Accordingly, ROS concentrations were markedly increased at least in IFN-β- and IFN-γ-treated CLL cells compared to control cells (Figure 1f). Taken as a whole, these results show that type I and II IFNs modulate the intrinsic apoptotic pathway and the mitochondrial activity in CLL cells.

### 3.2. Type I and II IFNs Mediate CLL Cell Survival through the STAT3/Mcl-1 Signaling Pathway

**Figure 2:**
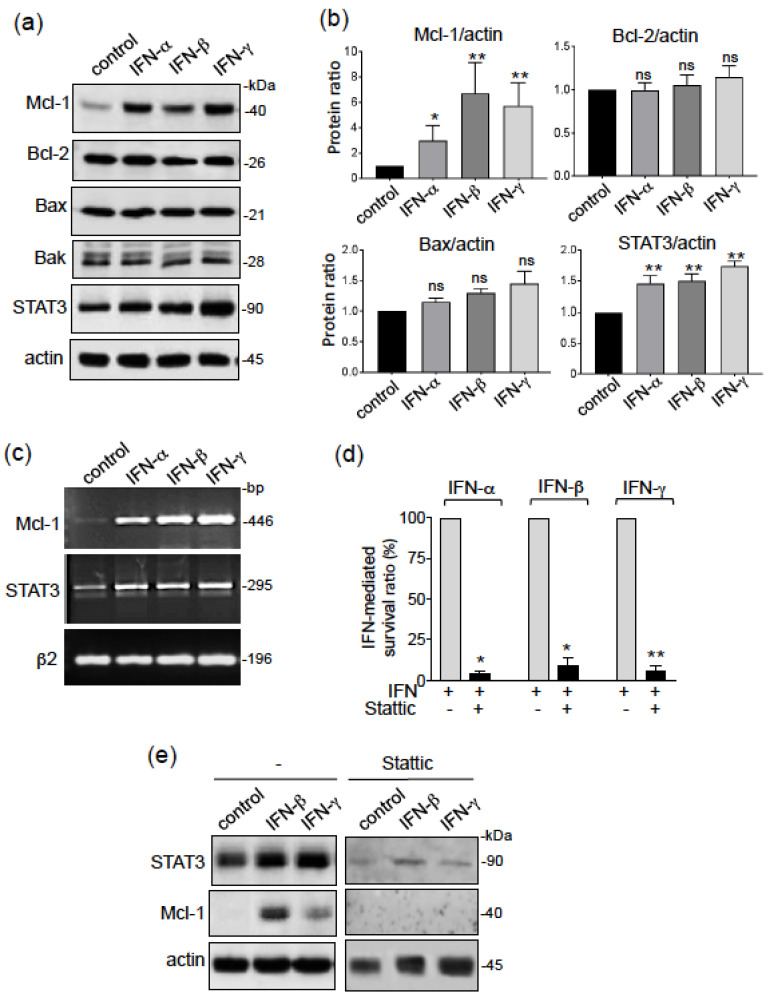
Type I and II IFNs promote CLL cell resistance to apoptosis through an STAT3/Mcl-1 signaling pathway. (**a**,**b**) Cell lysates from CLL cell samples cultured for 24 h in the presence or absence of IFNs (1000 U/mL), were examined for Mcl-1, Bcl-2, Bax, Bak, STAT3, and actin expression in immunoblot assays (reducing conditions). (**a**) A representative blot is shown; (**b**) Data (*n* = 4 for Bcl-2 and Bax; *n* = 6 for STAT3 and Mcl-1) are expressed as the ratio between the analyte proteins and actin, and presented as mean ± SEM; statistical relevance was assessed with the unpaired *t*-test. (**c**) RT-PCR analysis of Mcl-1, STAT3 and β2-microglobulin transcripts after CLL cell treatment for 18 h in the presence or absence of IFNs (1000 U/mL). (**d**,**e**) CLL cells were cultured for 24 h in the presence or absence of IFNs (1000 U/mL) after a 15 min pretreatment with 5 µM STAT3 inhibitor: (**d**) IFN-mediated cell survival was determined by subtracting the percentage of annexin-positive cells in the absence of IFN from the percentage of annexin-positive cells in the presence of IFN, and divided by the percentage of annexin-positive cells in the absence of stimulus × 100; the survival of IFN-treated cells in the absence of the inhibitor is considered as a ratio of 100%; the data are presented as mean ± SEM *(n* = 3). Statistical relevance was assessed with the paired *t*-test. (**e**) Western blots with antibodies against STAT3, Mcl-1 and actin. * *p* < 0.05; ** *p* < 0.01. Whole blots relative to the Western Blotting analyses are presented in Supplemental File Figure S1.

Mitochondrial membrane potential is influenced by the action of pro-apoptotic and/or anti-apoptotic members of the Bcl-2 family [39]. The anti-apoptotic proteins Bcl-2 and Mcl-1 are constitutively expressed in CLL cells, and are involved in the cells’ ability to prevent death [36]. The arrangement of pro-apoptotic protein Bax and Bak complexes in the mitochondrial membrane has a critical role in permeabilizing the outer mitochondrial membrane [42]. When activated by various stimuli (including type I/II IFNs), STAT3 can activate the gene expression of Mcl-1 and STAT3 itself, leading to the subsequent upregulation of Mcl-1 and STAT3 proteins [43,44]. We therefore used Western blots to analyze expression levels of Mcl-1, Bcl-2, Bax, Bak and STAT3 in CLL cells in the absence or presence of IFNs. After 24 h of culture, untreated cells expressed detectable levels of Mcl-1, Bcl-2, Bax, Bak and STAT3 (Figure 2a). Treatment with type I/II IFNs led to significant greater Mcl-1 levels (relative to untreated cells), whereas no significant differences were observed for levels of Bcl-2, Bax and Bak (Figure 2a). Activation of STAT3 through p^Y705^ is transient in CLL cells (from 5 min to 15 h) [45]. Accordingly, the relative levels of p^Y705^-STAT3 in CLL cells were found similar in IFN-treated cells and in untreated cells at 24 h of culture (data not shown), but the significant elevation in total STAT3 levels in all IFN-treated cells indicates that STAT3 was being induced (Figure 2a). The Mann–Whitney test confirmed the significant simultaneous upregulation of Mcl-1 and STAT3 in type I/II IFN-treated CLL cells (Figure 2b). In parallel, a representative RT-PCR assay showed that the transcripts of Mcl-1 and STAT3 were concomitantly enhanced in IFN-treated CLL cells, when compared with untreated cells (Figure 2c). Taken as a whole, these results strongly suggest that type I/II IFNs counteract mitochondrial-dependent CLL cell death by activating STAT3, which in turn upregulates the transcription of Mcl-1. We next treated CLL cells with Stattic, a selective STAT3 activation inhibitor that blocks the p^Y705^ in STAT3 and therefore prevents its binding to upstream kinases [46]. As shown in Figure 2d, treatment with Stattic (5 µM) almost totally counterbalances the enhancement of the viability mediated by IFNs in CLL cells. This inhibition was associated with the concomitant downregulation of STAT3 and Mcl-1 proteins (Figure 2e), indicating that the STAT3/Mcl-1 pathway was involved in the survival of CLL cells mediated by type I and II IFNs.

### 3.3. IFN-Mediated CLL Cell Survival and STAT3 Activation Involves Tyk2, JAK2 and Src Tyrosine Kinases

**Figure 3:**
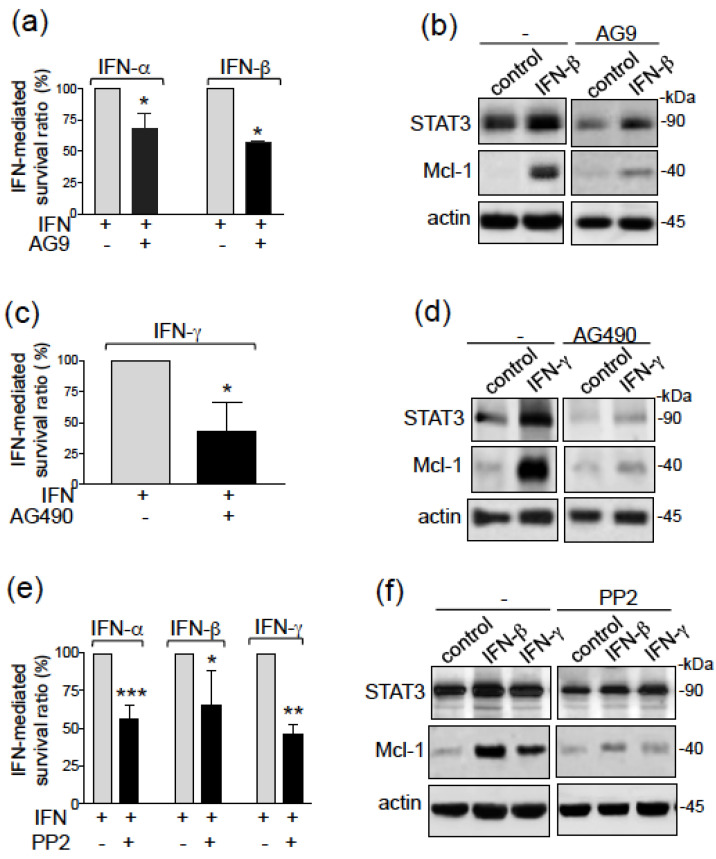
Type I and II IFNs induce CLL cell survival through the TYK2/Src and JAK2/Src signaling pathways respectively. (**a**–**f**) CLL cells were cultured for 24 h in the presence or absence of IFNs (1000 U/mL) after a 15 min pretreatment with (**a**,**b**) 10 µM AG9, (**c**,**d**)10 µM AG490 or (**e**,**f**) 10 μM PP2: (**a**,**c**,**e**) IFN-mediated cell survival was assessed as described in Figure 2; the survival of IFN-treated cells in the absence of the inhibitor is considered as a ratio of 100%; the data are presented as mean ± SEM (*n* = 2 for AG9; *n* = 4 for AG490; *n* = 4 for PP2); statistical relevance was assessed with the paired *t*-test. (**b**,**d**,**f**) Lysates were Western blotted (reducing conditions) with antibodies against STAT3, Mcl-1 and actin; representative experiments are shown. * *p* < 0.05; ** *p* < 0.01; and *** *p* < 0.001. Whole blots relative to the Western Blotting analyses are presented in Supplemental File Figure S1.

STAT3 can be activated by JAK2, TYK2 and Src kinases (c-Src, Fyn and Lyn) [5,6,32,47]. JAK2 is constitutively bound to the IFNGR2 chain of the IFN-γ receptor while TYK2 is bound to the IFNAR1 chain of the IFN-α/β receptors [2,6]. We therefore investigated which tyrosine kinase was involved in STAT3 activation leading to type I/II IFN-mediated CLL cell survival. To this end, we treated cells with AG9, a selective TYK2 inhibitor [48], AG490, a selective JAK2 inhibitor [49] and PP2, a Src family kinase inhibitor [50]. STAT3 activation and survival of IFN-α-treated CLL cells was previously shown to be prevented by AG9 [23]. In accordance, AG9 (10 µM) markedly blocked type I IFN-mediated CLL cell survival (Figure 3a) by downregulating the protein levels of STAT3 and Mcl-1 (Figure 3b). Treatment with AG490 (10 µM) inhibited the survival of CLL cells mediated by IFN-γ (Figure 3c) and this inhibition was associated with the downregulation of STAT3 and Mcl-1 proteins (Figure 3d). Finally, CLL cell survival mediated by type I and II IFNs was also significantly prevented by PP2 (10 μM) (Figure 3e) and accordingly, the relative levels of STAT3 and Mcl-1 proteins were downregulated in the presence of PP2 (Figure 3f). The combination of PP2 with AG9 or AG490 led to the induction of unspecific toxicity in CLL cells, making it impossible for assessing the additive effects of JAKs and Src inhibitors on IFN-mediated cell survival. Taken as a whole, these results indicate that type I and II IFNs promote the resistance of CLL cells to apoptosis by activating Src and TYK2 (IFN-α/β) or Src and JAK2 (IFN-γ) tyrosine kinases respectively, which in turn activate the survival STAT3/Mcl-1 axis.

## 4. Discussion

To the best of our knowledge, the effects and mechanisms of IFNs on CLL cell dysfunction have not previously been investigated in detail. We showed that both type I and II IFNs promote the survival of primary CLL cells by blocking spontaneous apoptosis mediated by the intrinsic mitochondrial pathway. Circulating IFNs are detected in serum from normal individuals [51,52], and elevated levels of serum IFN-γ are correlated with the advanced Rai stage disease [53,54]. Thus, it is possible that the concentrations of IFNs detected in vivo contribute to CLL survival and thus pathogenesis.

In IFN signaling, activation of TYK2 (for IFN-α and IFN-β) and JAK2 (for IFN-γ) leads to phosphorylation and homodimerization of STAT3, which is then translocated to the nucleus where it binds and activates the transcription of various genes including *MCL-1* and *STAT3* itself [5,6,43,44]. The anti-apoptotic Mcl-1 protein is an important regulator of the intrinsic mitochondrial pathway [55]. Resistance to the apoptosis of CLL B cells partly results from high expression of Mcl-1, which correlates with a poor prognosis and chemotherapy resistance [56,57]. Accordingly here, type I and II IFNs were found to upregulate the gene and protein expression of STAT3 and Mcl-1 in CLL cells. The results of our experiments with a specific STAT3 inhibitor indicate that type I/II IFN-mediated CLL cell survival involves the STAT3/Mcl-1 pathway. In CLL cells, under appropriate stimulation, STAT3 is either activated by TYK2 [23], JAK2 [58,59] or Lyn [45]. Here, we provide evidence that the IFNs’ effect on CLL cell survival is in part due to the activation of TYK2 (for type I IFN) or JAK2 (for type II IFN), leading to the sequential stimulation of STAT3 and Mcl-1. Treatment of IFN-treated CLL cells with JAK2 or TYK2 inhibitors did not completely abolish cell survival and upregulation of STAT3 and Mcl-1, suggesting the involvement of another tyrosine kinase in STAT3 activation. In this way, there is now evidence that activated cytokine receptors (including IFNs receptors) can stimulate Src family kinases involved in the full range of intracellular signaling events, including the tyrosine phosphorylation of STAT proteins [60,61]. In contrast to Fyn, Lyn is overexpressed in CLL cells and its inhibition with the Src inhibitor PP2 leads to the induction of death of leukemic cells [62]. Accordingly, our experiments with PP2 partly blocked IFN-mediated CLL cell survival and STAT3/Mcl-1 signaling. Interaction of promatrix metalloproteinase-9 (proMMP-9) to its receptors α4β1 integrin and CD44, induces CLL cell survival through an Lyn/STAT3/Mcl-1 signaling pathway [45]. It remains to be seen whether the effect of IFNs on CLL cell survival depends on Lyn activation.

It remains not clear how IFNs, following their binding to their specific receptors, can recruit and activate Src kinases [61]. Although c-Src, Fyn and Lyn are phosphorylated in response to IFN-γ treatment, JAKs’ kinase activity is not directly involved in the activation of these Src kinases, and none of these kinases directly interact with the IFN-γ receptor [61]. In tumor B cell lines, Fyn interacts with the activated forms of TYK2 and JAK2 in response to IFN-α or IFN-γ stimulation respectively [63]. Finally, in human NCI-H292 tumor epithelial cells, IFN-γ activates phospholipase C-γ (possibly via an upstream tyrosine kinase distinct from JAK1/JAK2) which induces the sequential activation of PKC-α c-Src and STAT1 [64]. Whether a similar scenario involving a phospholipase C-γ/PKC-α Src/STAT3 pathway occurs in CLL cells upon IFNs’ stimulation remains to be shown.

There is constitutive activation of BCR signaling in CLL [65,66]. The expression of BTK, a key component of proximal BCR signaling, is upregulated in CLL cells relative to non-malignant B cells, and targeting BTK in CLL with ibrutinib leads to direct cytotoxicity [65,66]. Src kinases (including Lyn and c-Src) can activate BTK [67]. One study suggested that STAT3 was involved as a BTK substrate [68]. We therefore wondered whether IFN-mediated STAT3 activation first activated an Src kinase and then BTK, leading to further STAT3 activation in CLL cells. Our preliminary experiments however showed that ibrutinib did not affect IFN-mediated CLL cell survival strongly suggesting that BTK is not involved in IFN signaling.

**Figure 4:**
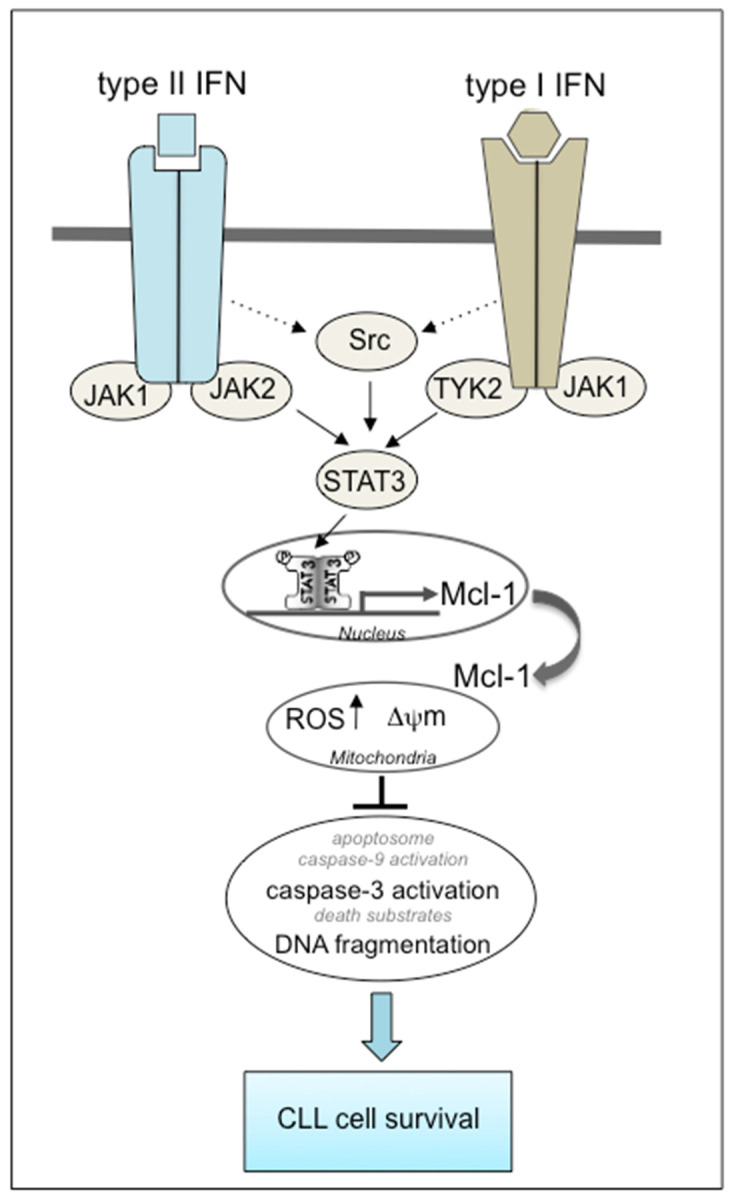
Putative model for the involvement of cell signaling pathways in the induction of survival by type I (α/β) and II (γ) IFNS in CLL cells. By binding to their respective IFN-receptors, type I and II IFNs likely lead to the activation of JAKs (TYK2 and JAK2 respectively) and an Src member kinase, which in turn activate STAT3. STAT3 dimer enters the nucleus and binds the *MCL1* promoter. Following *MCL1* transcription, Mcl-1 protein accumulates in the cytoplasm, and Mcl-1 exerts its anti-apoptotic activity by preventing mitochondrial depolarization, leading to the inhibition of caspase-3 activation and DNA fragmentation, ultimately favoring cell resistance to apoptosis. Both type I and II IFNs increase ROS mitochondrial concentrations, possibly through the sequential activation of STAT3 and its target gene LPL that shifts the metabolic program of CLL cells towards the utilization of lipids and then to an abundant ROS production into the mitochondria; increase 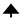.

Metabolic imbalances and augmented resistance to mitochondrial apoptosis are characteristics of CLL [40,69,70,71]. Two known master regulators of cell metabolism identified in CLL are STAT3 and miR-125 [70]. STAT3 activates the lipoprotein lipase (LPL) gene that shifts the metabolic program of CLL cells towards an abnormal fatty acid oxidation and then to an abundant ROS production into the mitochondria [70]. ROS appears to exhibit either pro- or anti-tumor effects in CLL [70]. The accumulation of ROS can facilitate apoptotic cell death [70]. Paradoxically, ROS promotes tumor progression by modifying the microenvironment and development of drug resistance in cancer cells [70,72]. In our study, concomitantly with cell survival, mitochondrial ROS concentrations are enhanced in IFN-treated CLL cells (Figure 4). As a whole, these data suggest that IFNs could also contribute to cell survival in CLL by activating an STAT3/LPL/ROS pathway.

Early studies showed that several cytokines and growth factors related to CLL pathogenesis, i.e., interleukin-6 (IL-6), vascular endothelial growth factor (VEGF), proMMP-9 and neutrophil gelatinase-associated lipocalin (NGAL), are implicated in CLL cell survival by activating the STAT3 (for VEGF and IL-6) and STAT3/Mcl-1 (for proMMP-9 and NGAL) signaling [45,73,74,75]. Therefore, it has to be considered whether IFN-treated CLL cells release these factors which, in an autocrine loop, activate STAT3. We previously showed that cultured CLL cells released no detectable to low levels of VEGF (0–7 pg/mL/10^6^ cells/48 h), IL-6 (0–40 pg/mL/10^6^ cells/48 h), and NGAL (0–19 ng/mL/10^6^ cells/48 h) [31,74], which were not altered by IFNs pretreatment [31] (B Bauvois, unpublished results). With regard to released proMMP-9 (158–1200 pg/mL/10^6^ cells/48 h), both type I and II IFNs suppressed the gene expression of proMMP-9 and its subsequent production [31]. These observations would indicate that none of these factors is involved in IFN-mediated CLL cell survival.

In summary, the results of the present study support the signaling model presented in Figure 4. This model indicates that type I and II IFNs promote the resistance to apoptosis of primary CLL cells through the simultaneous activation of TYK2 and Src, or JAK2 and Src kinases respectively, which in turn activate a STAT3/Mcl-1 signaling pathway, leading to the further modulation of both ΔΨ_m_ disruption, caspase-3 activation and DNA fragmentation.

## 5. Conclusions

The main treatments currently prescribed in CLL often lead to adverse drug reactions or favor drug resistance mutations [22,76,77]. An expected goal in CLL research is the development of therapeutic agents that block the expression/activity of targets which sustain the survival of malignant B cells. The inhibition of STAT3 or Mcl-1 could provide a therapeutic benefit by disrupting the survival STAT3/Mcl-1 axis in CLL cells. The development of potent small-molecule inhibitors specific for Mcl-1 have been reported in the literature [78,79]. There are now several Phase I clinical trials ongoing for hematological malignancies (including acute myeloid leukemia, non-Hodgkin’s lymphoma, myelodysplastic syndrome, myeloma multiple) [79], which are evaluating one inhibitor from Servier and Vernalis (R&D) Ltd. (S63415/MIK655), two from Amgen (AMG176 and AMG397) and one from AstraZeneca (AZD5991). Small-molecule STAT3 inhibitors of different STAT3 domains have been identified via the screening of chemical libraries and computational docking [11,12,80]. STAT3 inhibitors that target the SH2 domain of STAT3 (OPB-31121 and OPB-51602 from Otsuka Pharmaceuticals Co. Ltd.) have completed Phase I/II studies [12]. All these compounds are expected to progress in further clinical trials, and pave a new avenue for cancer therapy not only in the field of CLL but also in the general treatment of cancer.

## Figures and Tables

**Table 1:** Clinical characteristics of chronic lymphocytic leukemia (CLL) patients.

Characteristic	No (%)
**Total**	19 (100)
**Age, year** Median (range)	74 (57–85)
**Male**	10 (53)
**Binet stage** A B/C	14 (74) 5 (26)
**Lymphocytosis, G/L** Median (range)	46 (8–190)
**CD38 expression** >10%	/16 13 (81)
**Unmutated *IGHV***	6/11 (55)
**Karyotype** Normal 1–2 abnormalities Complex	/19 13 (68) 4 (21) 2 (11)
**FISH** Del(13q) Del(11q) Del(17p) Trisomy 12	/18 14 (74) 1 (5) 0 (0) 3 (16)

Abbreviations: G/L, giga per liter; *IGHV*, immunoglobulin heavy chain variable region; Del, deletion. One CLL patient was in relapse in 2017 following first-line treatment in 2015 with bendamustine and rituximab.

## Supplementary Materials

Supplementary materials can be found at https://www.mdpi.com/2227-9059/9/2/188/s1. Whole blots relative to the Western Blotting analyses.

## Abbreviations

CLL	chronic lymphocytic leukemia
IFN	interferon
STAT	signal transducer and activator of transcription
FCR	fludarabine-cyclophosphamide-rituximab
BCR	B cell receptor
BTK	Bruton’s tyrosine kinase
Bcl-2	B-cell lymphoma-2
PBMC	peripheral blood mononuclear cell
ROS	reactive oxygen species
LPL	lipoprotein lipase
