# Supplementary material for: Activation of Interferon Signaling in Chronic Lymphocytic Leukemia Cells Contributes to Apoptosis Resistance via a JAK-Src/STAT3/Mcl-1 Signaling Pathway
Source: Biomedicines. 2021 Feb 13;9(2):188. doi: 10.3390/biomedicines9020188 (PMC7918075; doi:10.3390/biomedicines9020188)
Supplement: Supplementary file 1 [file biomedicines-09-00188-s001.pdf]

Supplemental File Figure S1. Whole blots relative to the Western Blotting analyses

For Figure 2.A

Cell lysates from Patient 109: 2 Western Blots

Patient	Sex/Age	Stage	*IGHV* mutation	FISH	Karyotype	Therapy
109	M/75	B	UM	Trisomy 12	1 abnormality	None

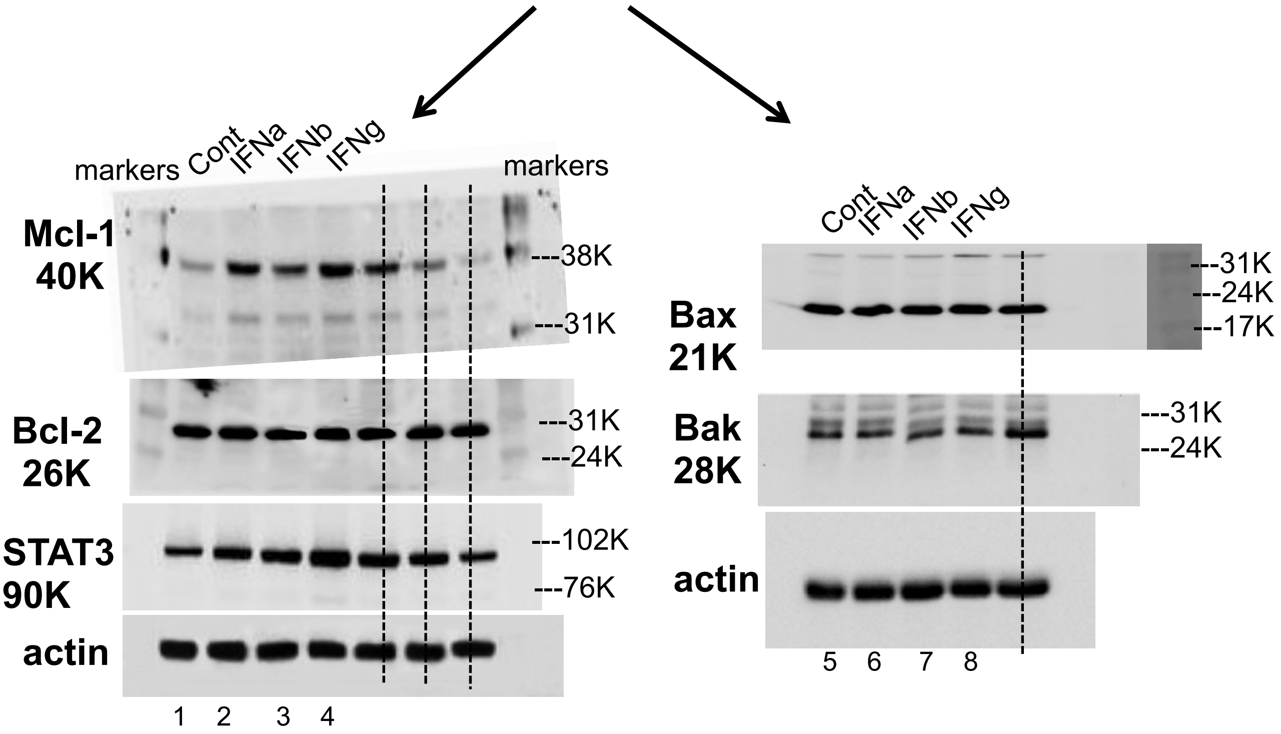

lane	1 control	2 + IFNa	3 + IFNb	4 + IFNg
Mcl-1/actin	1	2.2	1.7	2.8
Bcl-2/actin	1	1.0	0.8	0.9
STAT3/actin	1	1.3	1.5	2.2

lane	5 control	6 + IFNa	7 + IFNb	8 + IFNg
Bax/actin	1	1.1	1.1	1.2
Bak/actin	1	1.1	1.0	1.1

Control ratios were set to 1.0 to allow comparison between groups.

For Figure 2E
Cell lysates from Patient 126 : 2 Western Blots

Patient	Sex/Age	Stage	*IGHV* mutation	FISH	Karyotype	Therapy
126	M/76	C	ND	13q-	2 abnormalities	None

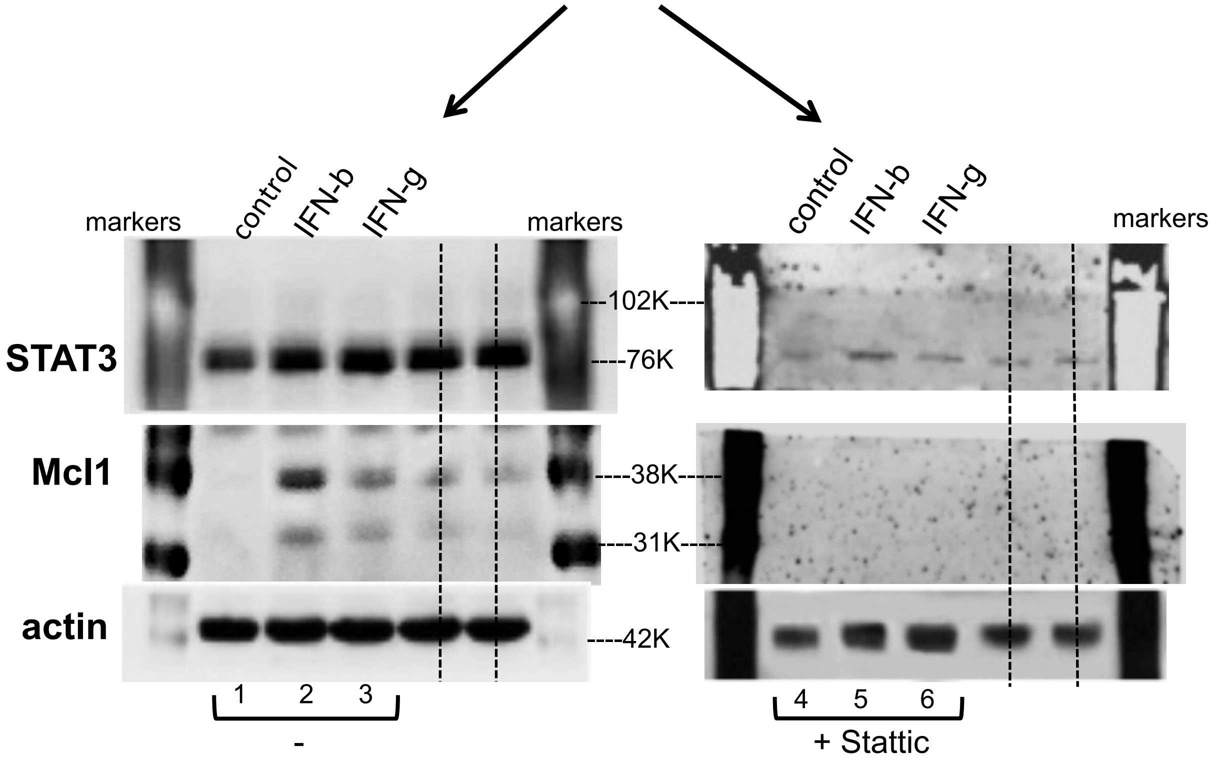

lane	1 control	2 + IFNb	3 + IFNg	4 control + Stattic	5 IFNb + Stattic	6 IFNg + Stattic
STAT3 /actin	1	2.2	2.3	1	1.3	1.1
Mcl-1/ actin	1	9.0	5.3	- --	---	---

Control ratios were set to 1.0 to allow comparison between groups.

For Figure 3B
Cell lysates from Patient 126 : 2 Western Blots

Patient	Sex/Age	Stage	*IGHV* mutation	FISH	Karyotype	Therapy
126	M/76	C	ND	13q-	2 abnormalities	None

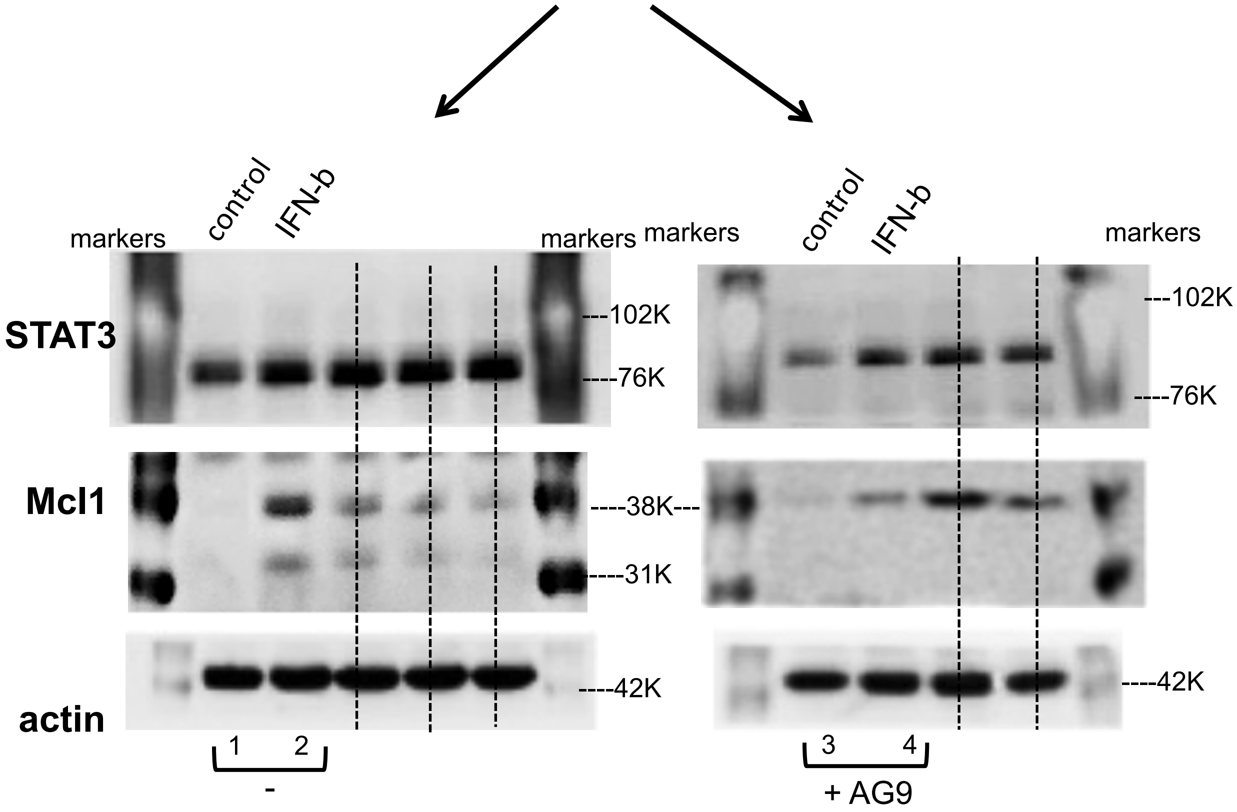

Lane	1 control	2 + IFNb	3 control + AG9	4 IFNb + AG9
STAT3/actin	1	2.2	1.0	1.5
Mcl1/actin	1	9.0	1.0	1.9

Control ratios were set to 1.0 to allow comparison between groups.

For Figure 3D
Cell lysates from Patient 128 : 1 Western Blot

Patient	Sex/Age	Stage	*IGHV* mutation	FISH	Karyotype	Therapy
128	M/71	A	M	Trisomy 12	2 abnormalities	None

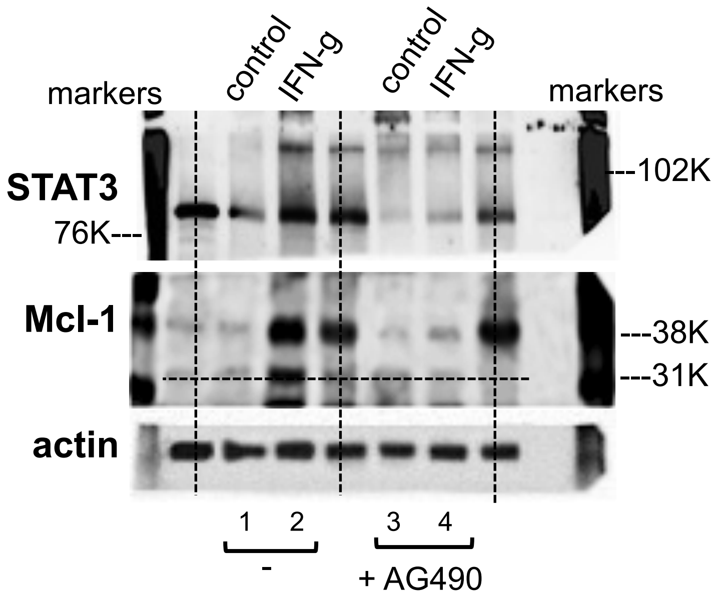

Lane	1 control	2 + IFNg	3 control + AG490	4 IFNg + AG490
STAT3/ actin	1	2.0	1.0	1.3
Mcl1/actin	1	6.2	1.0	1.8

Control ratios were set to 1.0 to allow comparison between groups.

For Figure 3F
Cell lysates from Patient 127 : 1 Western Blot

Patient	Sex/Age	Stage	*IGHV* mutation	FISH	Karyotype	Therapy
127	M/62	A	ND	Normal	Normal	None

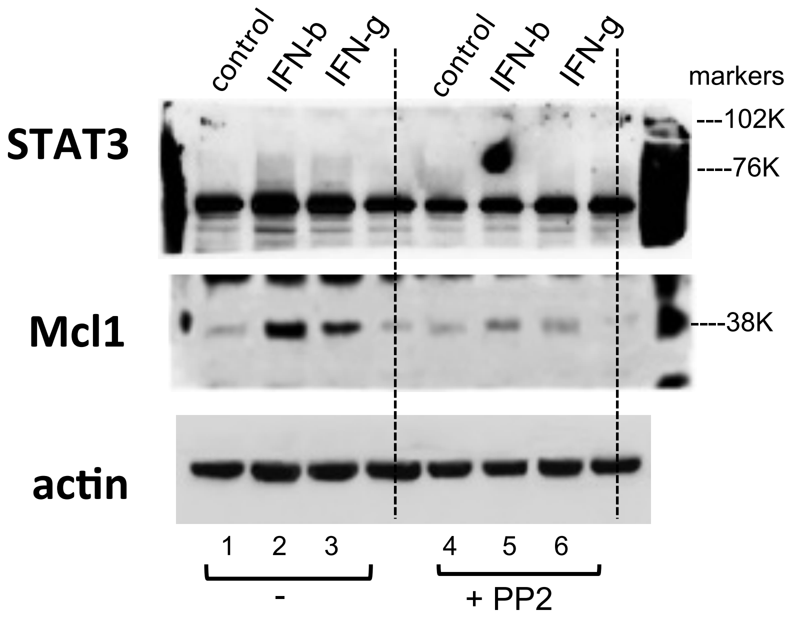

Lane	1 control	2 + IFNb	3 + IFNg	4 control + PP2	5 IFNb + PP2	6 IFNg + PP2
STAT3/ actin	1	1.6	1.6	1	1.2	1.1
Mcl-1/ actin	1	5.8	3.2	1	2.0	1.6

Control ratios were set to 1.0 to allow comparison between groups.
